# Disease progression modelling from preclinical Alzheimer’s disease (AD) to AD dementia

**DOI:** 10.1038/s41598-021-83585-3

**Published:** 2021-02-18

**Authors:** Soo Hyun Cho, Sookyoung Woo, Changsoo Kim, Hee Jin Kim, Hyemin Jang, Byeong C. Kim, Si Eun Kim, Seung Joo Kim, Jun Pyo Kim, Young Hee Jung, Samuel Lockhart, Rik Ossenkoppele, Susan Landau, Duk L. Na, Michael Weiner, Seonwoo Kim, Sang Won Seo

**Affiliations:** 1 Department of Neurology, Samsung Medical Center, Sungkyunkwan University School of Medicine, 81 Irwon-ro, Gangnam-gu, Seoul, 06351 South Korea; 2 Department of Neurology, Chonnam National University Medical School, Chonnam National University Hospital, Gwangju, South Korea; 3 Statistics and Data Center, Samsung Medical Center, 81 Irwon-ro, Gangnam-gu, Seoul, 06351 South Korea; 4 Department of Preventive Medicine, Yonsei University College of Medicine, Seoul, Korea; 5 Neuroscience Center, Samsung Medical Center, Seoul, South Korea; 6 Department of Neurology, Inje University College of Medicine, Haeundae Paik Hospital, Busan, South Korea; 7 Department of Neurology, Gyeongsang National University School of Medicine and Gyeongsang National University Changwon Hospital, Changwon, South Korea; 8 Department of Neurology, Myoungji Hospital, Hanyang University, Goyangsi, South Korea; 9 Internal Medicine-Gerontology and Geriatric Medicine, Wake Forest School of Medicine, Winston-Salem, NC USA; 10 Department of Neurology and Alzheimer Center, VU University Medical Center, Neuroscience Campus Amsterdam, Amsterdam, The Netherlands; 11 Helen Wills Neuroscience Institute, University of California, Berkeley, CA USA; 12 Stem Cell & Regenerative Medicine Institute, Samsung Medical Center, Seoul, South Korea; 13 Center for Imaging of Neurodegenerative Diseases, University of California, San Francisco, CA USA; 14 Department of Health Sciences and Technology, SAIHST, Sungkyunkwan University, Seoul, South Korea; 15 Samsung Alzheimer Research Center, Samsung Medical Center, Seoul, South Korea; 16 Department of Intelligent Precision Healthcare Convergence, Sungkyunkwan University School of Medicine, Suwon, South Korea

**Keywords:** Diseases, Neurology

## Abstract

To characterize the course of Alzheimer’s disease (AD) over a longer time interval, we aimed to construct a disease course model for the entire span of the disease using two separate cohorts ranging from preclinical AD to AD dementia. We modelled the progression course of 436 patients with AD continuum and investigated the effects of apolipoprotein E ε4 (APOE ε4) and sex on disease progression. To develop a model of progression from preclinical AD to AD dementia, we estimated Alzheimer’s Disease Assessment Scale-Cognitive Subscale 13 (ADAS-cog 13) scores. When calculated as the median of ADAS-cog 13 scores for each cohort, the estimated time from preclinical AD to MCI due to AD was 7.8 years and preclinical AD to AD dementia was 15.2 years. ADAS-cog 13 scores deteriorated most rapidly in women APOE ε4 carriers and most slowly in men APOE ε4 non-carriers (*p* < 0.001). Our results suggest that disease progression modelling from preclinical AD to AD dementia may help clinicians to estimate where patients are in the disease course and provide information on variation in the disease course by sex and APOE ε4 status.

## Introduction

Understanding the course of disease progression across the whole Alzheimer’s disease (AD) continuum including preclinical AD, mild cognitive impairment (MCI) due to AD, and AD dementia will help in designing clinical trials to test preventative interventions. Some studies have investigated the progression in preclinical AD^1^, MCI due to AD^2^ and AD dementia^3^ separately. However, their mean follow-up durations of 1.4–6.2 years were too short to understand the progression across the entire AD spectrum. Unfortunately, following a single cohort for several decades is difficult, though not impossible (as demonstrated in the Nun Study^4^, Framingham study^5^ etc.).

A potential approach would be to use cross-sectional and longitudinal data from many individuals across the disease spectrum from no AD pathology to AD dementia, to estimate a single disease progression model across. This method is advantageous, as it allows us to construct a disease course model for the whole-time span over a longer period using multiple separate cohorts. As far as we know, no such analysis has been used to the study of AD progression. Successfully constructing a model of the entire AD spectrum would allow an analysis of potential covariates that have been suggested to influence the disease process.

In the present study, we developed a model of AD progression across its entire spectrum using two separate cohorts. To investigate whether sex and APOE ε4 influence rates of cognitive decline across the AD continuum, we also constructed the disease models by sex and APOE ε4.

## Methods

### Participants

All data used in the present study were obtained from the Alzheimer’s Disease Neuroimaging Initiative (ADNI) website (http://www.adni-info.org) as of May 2017. ADNI is a multisite longitudinal biomarker study that has enrolled cognitively normal (CN), older individuals; people with early MCI (EMCI) and late MCI (LMCI) which are determined using the Wechsler Memory Scale Logical Memory II and people with early AD. EMCI defined as milder episodic memory impairment than the LMCI group. The present study consisted of 1091 participants enrolled in the ADNI-1, ADNI-GO and ADNI-2 cohorts who had available data for ADAS-cog13 testing and had ^18^F-AV45 (Florbetapir) PET to assess amyloid-β (Aβ) deposition. According to the National Institute on Aging-Alzheimer’s Association criteria^6–8^, Aβ (+) CN or subjective memory concerns (SMC) were defined as preclinical AD and Aβ (+) EMCI or LMCI were defined as MCI due to AD. In the present study, we included participants who were categorized as preclinical AD and MCI due to AD by their baseline diagnosis.

**Figure 1:**
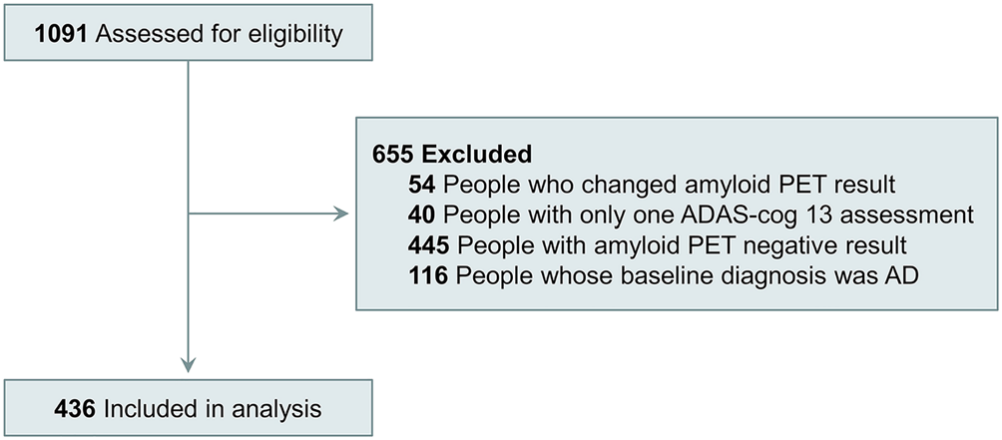
Flow diagram for selection of the study participants. We excluded the following participants: (1) 54 participants whose amyloid PET result changed from positive to negative; (2) 40 participants in whom ADAS-cog13 scores were obtained only once; (3) 445 participants with amyloid-negative PET results, because if amyloid-negative CN could become amyloid-positive, it would be difficult to create a disease progression model with amyloid-negative MCI; and (4) 116 participants with dementia at baseline, because their follow-up was short and the ADAS-cog 13 scores of AD dementia participants were in the range of the ADAS-cog 13 scores of participants who progressed from MCI due to AD to AD dementia. ADAS-cog: Alzheimer's Disease Assessment Scale-cognitive subscale, CN: cognitive normal; MCI: mild cognitive impairment; AD: Alzheimer's Disease.

We excluded the following conditions: (1) 54 participants whose amyloid PET result changes; their amyloid PET result changed from positive to negative. (2) 40 participants in whom the ADAS-cog13 scores were obtained only once. Therefore, all enrolled participants performed ADAS-cog 13 at least two times. (3) 445 participants with amyloid pet negative result because amyloid negative CN could become amyloid positive then it is hard to make disease progression model with amyloid negative MCI. (4) 116 participants with dementia at baseline were not included because their median time of follow-up was short (12 months) and ADAS-cog 13 scores for AD dementia participant were in the range of ADAS-cog 13 scores for participants who progressed from MCI due to AD to AD dementia (Fig. 1)^9^.

All participants signed written informed consent at the time of enrolment. The authors obtained approval from the ADNI Data Sharing and Publications Committee for data use and publication. Since all the analyses were performed using de-identified ADNI data which is available for download, no IRB review was required. All methods were carried out in accordance with the approved guidelines.

### Neuropsychological evaluation

For neuropsychological testing, participants undergo ADAS-Cog 13 at baseline, 6, 12, and ongoing annually performed for CN, MCI participants. We used ADAS-cog 13, which includes tests of attention and concentration, planning and executive function, verbal memory, nonverbal memory, praxis, delayed word recall, and number cancellation or maze tasks. ADAS-cog 13 scores range from 0 to 85. The ADAS-cog 13 is more responsive to disease progression than the ADAS-cog 11 in subjects with AD and similar or slightly more responsive in subjects with pre-dementia syndromes^10,11^.

### Image acquisition and processing

We downloaded amyloid (florbetapir) PET data from the ADNI website. Florbetapir imaging consisted of four 5-min frames (dynamic 3D scan) acquired 50–70 min after injection of 370 MBq (10 mCi) of tracer; frames were realigned, averaged, resliced to a common voxel size (1.5 mm × 1.5 mm × 1.5 mm) and smoothed to a common resolution of 8 mm^3^. MPRAGE images acquired concurrently with baseline florbetapir images and used as a structural template to define cortical and reference regions in native space for each subject with FreeSurfer. More detailed information can be found at http://www.loni.ucla.edu. A florbetapir cortical summary measurement (SUVR) was calculated by dividing cortical uptake by a whole cerebellum as a reference region. We included only amyloid-positive individuals with an Amyloid SUVR of 1.11^12^ or higher in our analysis.

### APOE genotyping

APOE genotyping was performed on DNA obtained from participant blood samples with an APOE genotyping kit as described at the ADNI site (see http://www.adni-info.org for detailed information on blood sample collection, DNA preparation, and genotyping methods). APOE ε4 non-carriers were defined as no APOE ε4 allele and APOE ε4 carriers as one or two APOE ε4 alleles.

### Statistical analysis

**Figure 2:**
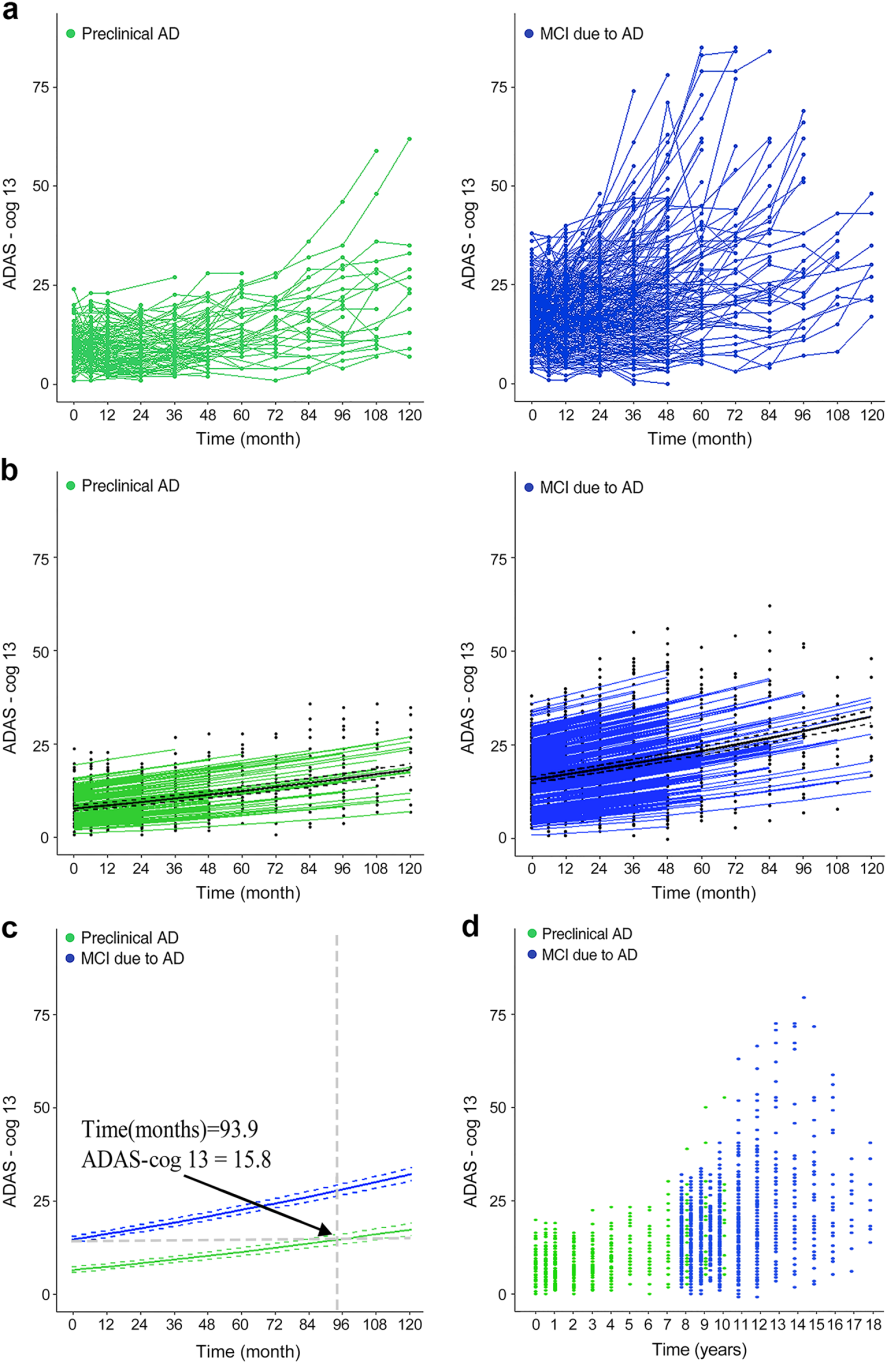
Modelling the course of Alzheimer’s disease using ADAS-cog 13 scores. (**a**) The pattern of individual ADAS-cog 13 scores in individuals with preclinical AD and MCI due to AD. (**b**) The estimated ADAS-cog 13 scores over time for each subject and for each cohort, obtained from a linear mixed effects model with time as a fixed effect and subjects as a random effect (excluding outliers). Green and blue lines mean the estimated ADAS-cog 13 score for each subject at the time for preclinical AD and MCI due to AD, respectively. Black solid and dotted lines mean the estimate and 95% confidence interval (CI) for mean ADAS-cog 13 score at the time. (**c**) The estimated mean ADAS-cog 13 score and the corresponding time for two cohorts to start to overlap. Solid line means the estimated mean ADAS-cog 13 score and dotted lines mean 95% CI of the estimated mean ADAS-cog 13 score. (**d**) Scatter plot of the combined preclinical AD and MCI due to AD cohorts shifted by the time of 93.9 months, corresponding to an ADAS-cog 13 score of 15.8 points. ADAS-cog: Alzheimer's Disease Assessment Scale-cognitive subscale, MCI: mild cognitive impairment; AD: Alzheimer's Disease.

**Figure 3:**
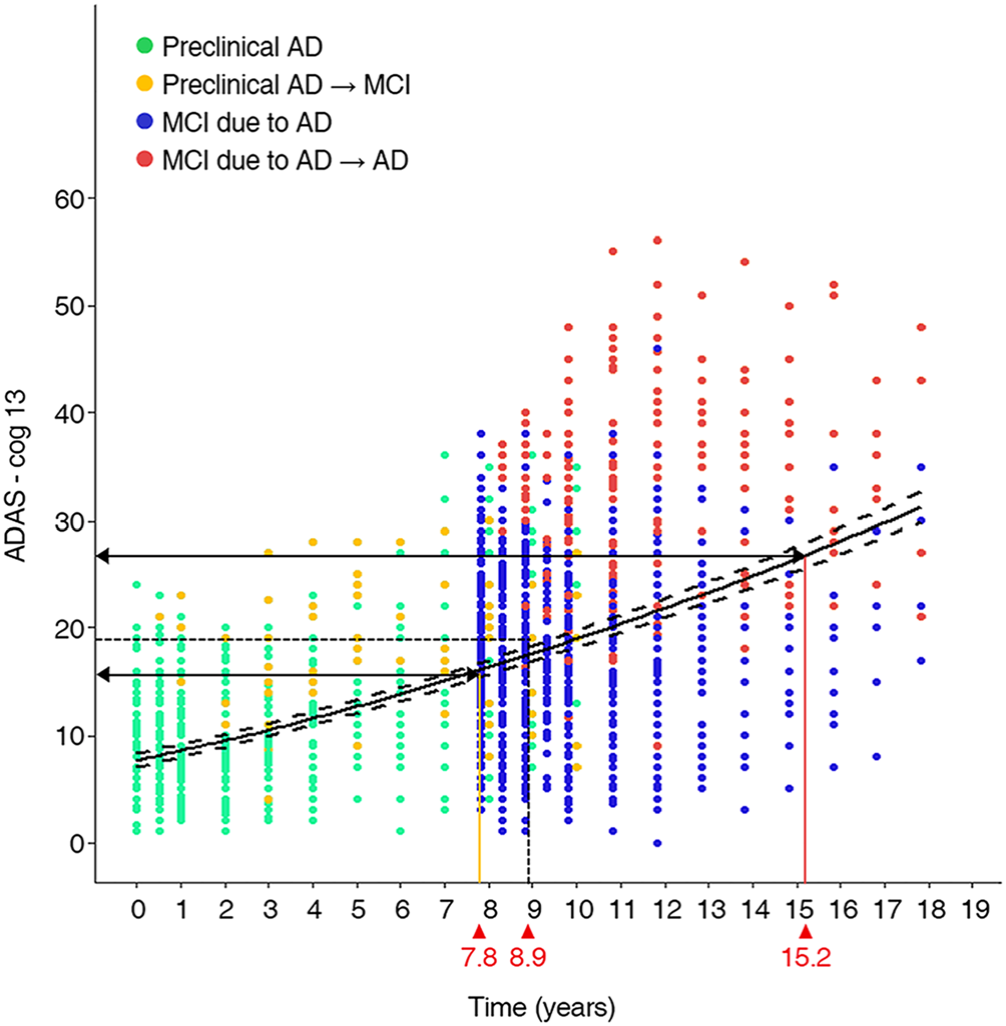
Disease progression model from preclinical AD to AD dementia. The curves present the estimated model—ADAS-cog 13 = (2.8492 + 0.0130 × month)^2^ – 0.5 and its 95% CI and the plots show preclinical AD (green dots), progression to MCI due to AD (yellow dots), MCI due to AD (blue dots), and progression to AD dementia (red dots). Using the median ADAS-cog 13 scores at the time of progression for individuals who progressed from preclinical AD to MCI due to AD (16.0 points) and from MCI due to AD to AD dementia (26.8 points), we estimated the time for preclinical AD to progress to MCI due to AD (7.8 years) and to AD dementia (15.2 years). When using the median ADAS-cog 13 scores for late MCI (19.0 points) to estimate time to progression, it took 8.9 years for preclinical AD to progress to late MCI. ADAS-cog: Alzheimer's Disease Assessment Scale-cognitive subscale; MCI: mild cognitive impairment; AD**:** Alzheimer's Disease; CI: Confidence interval.

**Figure 4:**
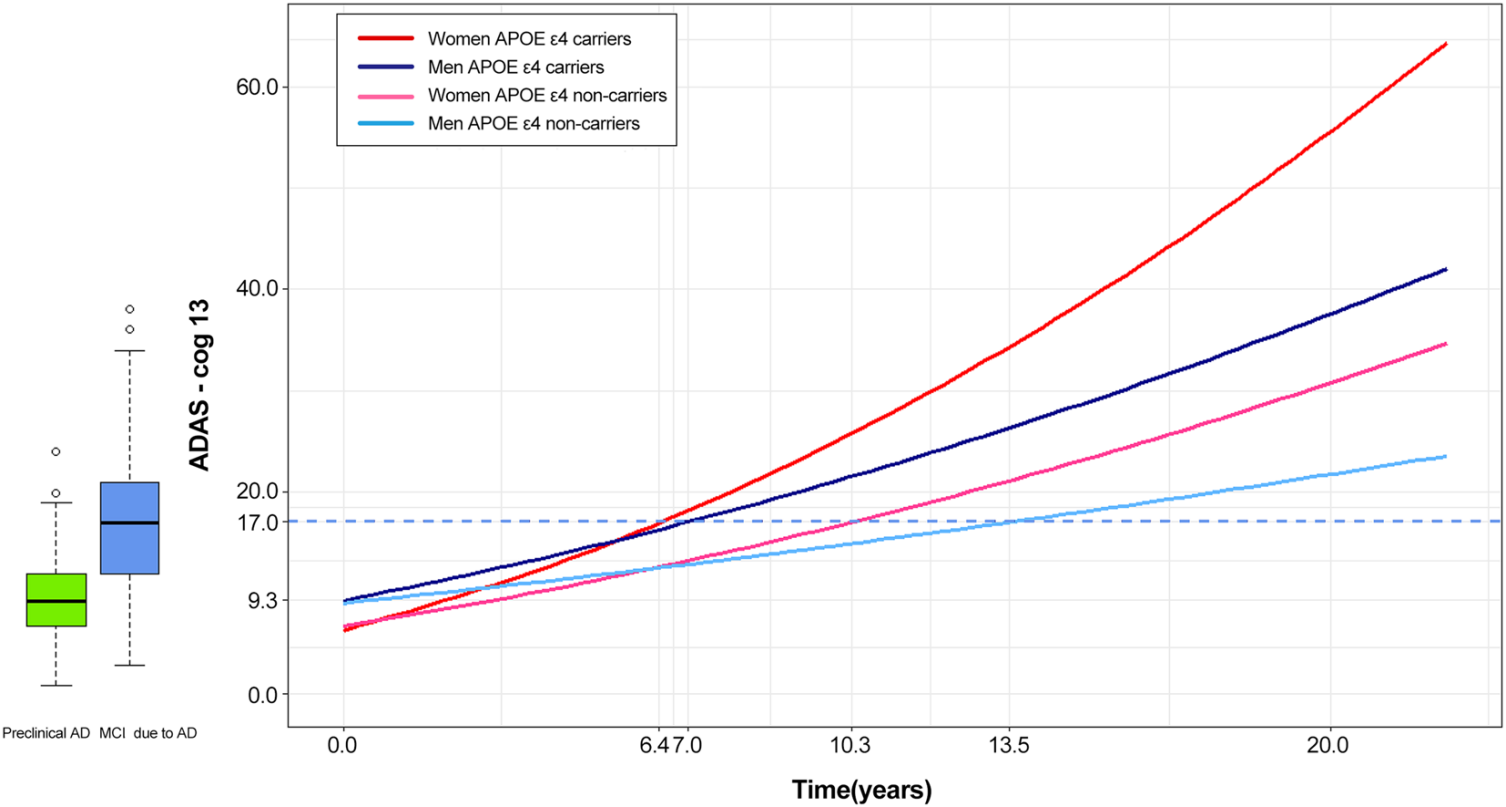
Sex and APOE ε4 effects on disease progression**.** We analysed differences in cognitive decline by sex and APOE ε4 status. Different-coloured lines indicate women APOE ε4 carriers (red), women APOE ε4 non-carriers (pink), men APOE ε4 carriers (dark blue) or men APOE ε4 non-carriers (light blue). The box plot shows the median value of ADAS-cog 13 was 9.3 for preclinical AD and 17.0 for MCI due to AD. The time differences between APOE ε4 carriers and non-carriers at baseline median ADAS-cog 13 in the MCI due to AD cohort (17.0 points) were 3.9 years for women (10.3 years (APOE ε4 non-carriers)—6.4 years (APOE ε4 carriers)) and 6.5 years for men (13.5 years (APOE ε4 non-carriers)—7.0 years (APOE ε4 carriers)). The estimated equation for each sex and APOE ε4 combination is as follows: ADAS Cog-13 = (2.6131 + 0.0203 × month)^2^ – 0.5 for women APOE ε4 carriers, = (2.6842 + 0.0121 × month)^2^ – 0.5 for women APOE ε4 non-carriers, = (3.1198 + 0.0127 × month)^2^ – 0.5 for men APOE ε4 carriers, = (3.0806 + 0.0068 × month)^2^ – 0.5 for men APOE ε4 non-carriers. ADAS-cog: Alzheimer's Disease Assessment Scale-cognitive subscale; APOE: Apolipoprotein E.

In order to model the disease progression course from preclinical AD to AD dementia using two cohorts, we carried out the following three processes: (1) modelling of ADAS-cog 13 scores for each cohort, (2) calculating the time for ADAS-cog 13 scores from the two cohorts to start to overlap, (3) constructing an entire disease continuum model. First, for the estimation of the model for longitudinal data (Fig. 2a), the mixed-effects model with a random effect for the subject and a fixed effect for time was applied to each set of disease cohort data. In the development of the model, ADAS-cog 13 scores were square root–transformed due to a highly skewed distribution, and outliers with an absolute studentized residual larger than 3 were excluded (Fig. 2b). Second, using the estimated mixed effect model for each cohort, the estimate and 95% confidence interval (CI) for mean ADAS-cog 13 scores at the time were calculated. If a point estimate of mean ADAS-cog 13 score in MCI due to AD fell within the 95% CI of mean ADAS-cog 13 scores in preclinical AD, that a mean estimate was considered as an overlapped mean ADAS-cog 13 score between the two cohorts. We found the smallest mean score among the overlapped mean ADAS-cog 13 scores in the MCI due to AD cohort and substituted this mean score into the estimated model for the preclinical AD cohort to calculate the corresponding time to this mean ADAS-cog13 score. This indicated the time from mean baseline ADAS13 for preclinical AD to mean baseline ADAS13 for MCI due to AD (Fig. 2c). Then, we shifted the MCI due to AD cohort data to start from that time (Fig. 2d). Finally, a single model for the entire course of AD was estimated by analysing data from the second step using a linear mixed effects model that included the same effect terms as the individual cohort models. In this model, the duration and its 95% CI for progression from preclinical AD to MCI due to AD and to AD dementia was calculated in terms of the time corresponding to the median ADAS-cog 13 scores and the 95% CI for the progressed groups (Fig. 3). To investigate the effect of sex and APOE ε4 status on ADAS-cog 13 decline, another progression model for the entire AD continuum was developed using a linear mixed effects model that included the combined effects of sex and APOE ε4 carrier status, as well as a time effect and a random intercept effect (Fig. 4). Model fit was investigated using the Akaike information criterion (AIC), Bayesian information criterion (BIC), and AIC with correction for finite sample size (AICC).

In a sensitivity analysis, we examined the learning effect and the effect of the different APOE ε4 allele distributions between the cohorts on the estimated results. We adjusted for learning effects (LEs), because LEs related to repeated measurements may obscure cognitive decline and delay the detection of conversion to MCI^13^ and AD. The magnitude of LEs was estimated and tested with six alternative linear mixed models according to the covariates of age at baseline, sex, and education level^14^. ADAS-cog 13 scores adjusted for LEs were used for the sensitivity analysis. We also performed frequency matching of APOE ε4 allele carriage for preclinical AD and MCI due to AD, and estimated the ADAS-cog 13 score and the corresponding time for the two cohorts to start to overlap using the matched data.

*P*-values were corrected for multiple testing using the Bonferroni method. Continuous and categorical variables were summarized as median (inter-quartile range (IQR, 1st quartile–3rd quartile) and frequency (percentage), respectively. A two-tailed *P*-value < 0.05 was considered to indicate statistical significance. The statistical analysis was performed with SAS 9.1.3 (SAS Institute Inc, Cary, NC, USA) and the R3.4.1 package (Vienna, Austria).

## Results

### Demographic and clinical characteristics of participants

The preclinical AD cohort included 127 participants, while the MCI due to AD cohort included 309 participants (Table 1). The median age of participants with preclinical AD was 74.6 years (IQR 70.8–78.5), while that of participants with MCI due to AD was 73.6 years (68.5–78.1). In the preclinical AD cohort and in the MCI due to AD cohort, 57 participants (44.9%) and 210 participants (68.0%) were APOE ε4 carriers, respectively. Women comprised 79 participants (62.2%) in the preclinical AD cohort, and 130 (42.1%) in the MCI due to AD cohort. The median years of education were 16 for both the preclinical AD cohort and the MCI due to AD cohort. The number of visits (median (IQR)) per participant was 5 (3–7) in the preclinical AD cohort and 6 (4–7) in the MCI due to AD cohort. The follow-up period was 48 (24–72) months in the preclinical AD cohort and 48 (36–60) months in the MCI due to AD cohort. The median (IQR) ADAS-cog 13 scores were 9.3 (6.7–12.0) in the preclinical AD cohort and 17 (12.0–21.0) in the MCI due to AD cohort. In the preclinical AD cohort, 37 participants (29.1%) progressed to MCI due to AD and 13 (10.2%) progressed to AD dementia. In the MCI due to AD cohort, 134 participants (43.4%) progressed to AD dementia.

**Table 1:** Demographics and clinical features of participants with AD.

Diagnosis	Preclinical AD	MCI due to AD
Participants no. (%)	127 (29.2)	309 (70.9)
Age (year), median (IQR)	74.6 (70.8–78.5)	73.6 (68.5–78.1)
APOE ε4 carriers, no. (%)	57 (44.9)*	210 (68.0)*
Women, no. (%)	79 (62.2)*	130 (42.1)*
Education (year), median (IQR)	16 (14–18)	16 (14–18)
**Follow up**		
Number of visits per participant, median (IQR)	5 (3–7)	6 (4–7)
Follow up month, median (IQR)	48 (24–72)	48 (36–60)
**ADAS-cog 13**		
Median (IQR)	9.3 (6.7–12.0)*	17 (12.0–21.0)*
**Conversion to**		
MCI due to AD, no (%)	37 (29.1)	
AD dementia, no (%)	13 (10.2)	134 (43.4)

Age, education, ADAS-cog 13 and month of follow-up are expressed as median (IQR).

Categorical variables are expressed as no (%). Statistical analyses are performed with Chi-squared tests for APOEε4 carriers and sex. Mann Whitney test for age, education and ADAS-cog 13.

AD: Alzheimer's disease; ADAS-cog: Alzheimer's disease assessment scale-cognitive subscale; APOE: apolipoprotein E; IQR: interquartile range; MCI: mild cognitive impairment.

*p < 0.05 between preclinical AD vs. MCI due to AD.

### Disease progression modelling from preclinical AD to AD dementia

The median ADAS-cog 13 score was 16.0 points at the time of progression for participants who progressed from preclinical AD to MCI due to AD and 26.8 points at the time of progression for participants who progressed from MCI due to AD to AD dementia. The estimated years (95% CI) for progression from the median ADAS-cog 13 score in the preclinical AD cohort (9.3 points) to the median ADAS-cog 13 at the time of progression in participants who progressed from preclinical AD to MCI due to AD (16.0 points) was 7.8 (6.1–10.0) years. The estimated years for progression from preclinical AD to the median ADAS-cog 13 at the time of progression in participants who progressed from MCI due to AD to AD dementia (26.8 points) was 15.2 (14.1–15.9) years (Fig. 3). Additionally, when the calculation was performed using the median ADAS-cog 13 score for LMCI (19 points), the estimated time to progress from preclinical AD to LMCI was 8.9 years.

### APOE ε4 effects on the course of disease progression by sex

We analysed the individual effect of sex and APOE ε4 on ADAS-cog 13 score change over time for each cohort. APOE ε4 carriers had a faster decline in ADAS-cog 13 score than APOE ε4 non-carriers in both cohorts (*p* = 0.0036 for preclinical AD, *p* < 0.0001 for MCI due to AD). Women had a steeper decline in ADAS-cog 13 score than men (*p* < 0.0001 for both cohorts). Then, to discover the combined effect of sex and APOE ε4 in the AD continuum, we analysed differences in the rate of cognitive decline stratified by sex and APOE ε4 status (Fig. 4). APOE ε4 carriers had a steeper decline in ADAS-cog 13 scores than did APOE ε4 non-carriers regardless of sex (*p* < 0.001). Women also had a steeper decline in ADAS-cog 13 scores than men, irrespective of APOE ε4 carrier status (*p* < 0.001). ADAS-cog 13 scores deteriorated most rapidly for women APOE ε4 carriers and most slowly for men APOE ε4 non-carriers (*p* < 0.001). Using the median ADAS-cog13 values for participants with MCI due to AD who progressed to AD dementia, we calculated the time to progress from preclinical AD to AD dementia for four combinations of sex and APOE ε4 status (Table 2). We estimated that women APOE ε4 carriers with a median ADAS-cog 13 score (29 points) at the time of progression would take 11.5 (95% CI 10.0–11.9) years to progress to AD dementia. When estimated in the same way, men APOE ε4 carriers took 12.7 (10.5–14.0) years to progress from preclinical AD to AD dementia, while women APOE ε4 non-carriers took 20.2 (13.5–23.7) years and men APOE ε4 non-carriers took 24.0 (17.7–30.9) years. In our disease model, we found that there were time differences between APOE ε4 carriers and non-carriers in baseline median ADAS-cog 13 in the MCI due to AD cohort: 3.9 years for women and 6.5 years for men (Fig. 4). More importantly, this difference started at the baseline median ADAS-cog13 score for the preclinical AD cohort. To discover which model fit the data best, we performed goodness of fit test for models with and without sex and APOE ε4 (Supplementary Table S1). The model including sex and APOE ε4 was better than the model without those variables.

**Table 2:** Estimated time to reach AD dementia depending on APOE ε4 status by sex.

Group	Participants (N)	ADAS-cog 13^a^ Median (IQR)	Estimated years^b^ (95% CI)
Women APOE ε4 carriers	44	29 (23.5–33.0)	11.5 (10.0–11.9)
Men APOE ε4 carriers	57	25 (20.0–31.0)	12.7 (10.5–14.0)
Women APOE ε4 non-carriers	14	31 (23.0–34.0)	20.2 (13.5–23.7)
Men APOE ε4 non-carriers	19	25 (20.0–31.0)	24.0 (17.7–30.9)

We assigned the median ADAS-cog 13 score at the point of conversion from MCI due to AD to AD dementia to the equation for each sex and APOE ε4 combination to obtain the estimated converting year to AD dementia.

ADAS-cog: Alzheimer's disease assessment scale-cognitive subscale; APOE: apolipoprotein E; IQR: interquartile range; CI: confidence interval.

^a^ADAS-cog 13 median (IQR) at the point of conversion from MCI due to AD to AD dementia.

^b^Estimated years from preclinical AD to AD dementia.

### Sensitivity analysis

We performed frequency matching for APOE ε4 allele carriage between preclinical AD and MCI due to AD cohort, and calculated the time for a subject to convert from the preclinical to prodromal stage to examine the effect of the different APOE4 ε4 allele distributions between preclinical AD and MCI due to AD cohorts on the estimated results (Supplementary Fig. S1). The matched data showed ADAS-cog 13 score for the two cohorts at start of overlap was estimated as 15.1 (95% CI 14.1–16.2) and the corresponding time was 7.4 years (88.2 months, 95% CI 77.0–99.4).This result did not differ much from the result using the unmatched data (7.8 years, Fig. 3), but the time at which the two cohorts began to overlap was a little shorter in the matched data.

Additionally, we performed an analysis for LEs to investigate the robustness of LEs. LEs were significant and were estimated to affect a given ADAS-cog 13 score by − 0.52 for preclinical AD and by − 0.54 for MCI due to AD in all models (Supplementary Table S2). After correcting for LEs and repeating the analyses, we estimated the disease progression course from preclinical AD to AD dementia according to ADAS-cog 13 scores. The estimated times for preclinical AD to progress to MCI due to AD and to AD dementia were 6.7 (95% CI, 5.0–9.0) and 14.2 (13.1–14.9) years based on median ADAS-cog 13 scores (supplementary Fig. S2). When we analysed differences in the rate of cognitive decline based on a combination of sex and APOE ε4 status after correcting for LEs (supplementary Fig. S3), the progression order and significant differences among groups did not change compared to the analysis of data uncorrected for LE.

## Discussion

In the present study, using two separate cohorts, we modelled disease progression from preclinical AD to AD dementia and determined whether APOE ε4 status and sex affected progression across the entire AD spectrum. Our main findings were as follows. Our novel disease progression model indicated that it would take 7.8 years for preclinical AD to progress to MCI due to AD and 15.2 years to progress to AD dementia based on median ADAS-cog 13 scores. APOE ε4 carriers and women had worse cognitive trajectories across the entire AD spectrum. Across all sex and APOE ε4 combinations, women APOE ε4 carriers had the fastest cognitive decline. Taken together, our findings provide a further understanding of AD progression across the disease spectrum, and they will help to design individualized therapeutic and preventive strategies to ameliorate cognitive decline.

We modelled the AD disease progression course using two different cohorts and estimated that it took almost 15 years for preclinical AD to progress to AD dementia. In a recent article^15^, 14.5% of individuals with preclinical AD developed incident MCI due to AD within a 3.7 year (mean) follow-up period, and 3.2% developed AD dementia within 4.2 years of follow-up^15^. Additionally, studies have found that 32.7%^15^ and 70.0%^16^ of individuals with MCI due to AD developed AD dementia within 3.2 and 3.6 years of follow-up, respectively^15,16^. However, 2–4 years of follow-up may not be sufficient to estimate the entire course of disease progression. These previous findings, thus, mainly characterize fast decliners in each disease stage. However, our estimated course is consistent with indirect evidence provided in previous studies^6,17^, according to which the temporal lag between Aβ deposition and the clinical syndrome of AD dementia was a decade^6^. In a meta-analysis, age-related increases in amyloid positivity on PET in participants with normal cognition paralleled age-specific, AD-type dementia prevalence estimates with an intervening period of about 20 years^17,18^. Another study estimated that it took 19.2 years for ^11^C-PiB levels observed in healthy controls with a 1.5 SUVR threshold to reach the mean SUVR of AD (2.3)^19^. Our finding that it would take more than 15 years for preclinical AD to progress to AD dementia suggests that appropriate interventions are needed to prevent preclinical AD from progressing to AD dementia.

In the present study, the estimated time from the preclinical to prodromal stage (7.8 years) was similar to that from the prodromal to the dementia stage (7.4 years). Initially, we expected that the preclinical phase might be longer than the prodromal phase. Our findings might have been related to our definition of the prodromal phase using the early stage of MCI. If we define MCI due to AD as LMCI, the estimated time from preclinical AD to LMCI (8.9 years) would be longer than that from MCI due to AD to AD dementia (6.3 years). Alternatively, the study design—in particular, whether a study includes volunteer or clinic-based participants—might affect time-to-event estimates. For example, studies may overestimate the progression rate in the presymptomatic phase because the included participants might have more concerns about their cognition. Our disease progression model could be used to estimate the current and future state of preclinical AD patients in a prevention trial.

Another main finding is that sex and APOE ε4 had distinct effects on the progression course across the AD continuum. Our finding that APOE ε4 aggravated cognitive decline across the entire AD spectrum regardless of sex is partially consistent with previous studies. While APOE ε4 is a well-known risk factor for AD dementia in the preclinical or prodromal stage^20^, it has been debated whether APOE ε4 predicts a worse prognosis^21,22^. A previous study by our group revealed that APOE ε4 predicted more rapid hippocampal and cortical atrophy in dementia with AD^21^. However, other studies have suggested that AD patients with APOE ε4 had a lower global amyloid burden than matched APOE ε4 non-carriers^22–24^. This discrepancy might be due to differences in the study populations (patients who progressed to AD dementia over time in the current study sample compared to patients who had already progressed to AD dementia in previous studies).

A more noteworthy finding that women APOE ε4 carriers showed more prominent cognitive decline than did men APOE ε4 carriers across the AD spectrum^25,26^. Our findings are consistent with a previous study^25^, which showed that women with higher Aβ levels had a faster cognitive decline than men and that women with preclinical AD who were APOE ε4 carriers declined faster than their men counterparts. However, the previous findings were not statistically significant after correction for multiple comparisons^25^. Our findings further suggest that women APOE ε4 carriers had a steeper cognitive decline than did men APOE ε4 carriers throughout the entire AD spectrum. Therefore, developing a progression model stratified by these factors will help to select cohorts for AD clinical trials.

Several possible explanations may account for the combined effects of sex and APOE^27–30^. A potential mechanism could be that oestradiol promotes synaptic sprouting in response to injury through an APOE-dependent mechanism^27^. Additionally, oestrogen might promote neural function under normal conditions, but exacerbate dysfunction when network activity is disrupted^28^. Alternatively, a previous study showed that the APOE ε4-by-sex interaction on cerebrospinal fluid (CSF) tau levels were significant, suggesting that the increased APOE-related risk in women may be associated with tau pathology^29^. In a recent multicohort study^30^, women showed a stronger association between APOE and CSF tau levels than did men, particularly among amyloid-positive individuals, suggesting that APOE may modulate the risk of downstream neurodegeneration in a sex-specific manner, particularly in the presence of amyloidosis.

The ADNI is a well-organized, longitudinal cohort that serves as an excellent resource to investigate the disease course of AD. This study, however, has several limitations. We only included participants who were amyloid-positive by PET. This leaves open the possibility that some patients had another primary pathological diagnosis. Although participants clinically diagnosed with frontotemporal dementia or dementia with Lewy bodies and who had moderate to severe white matter hyperintensity were excluded from the ADNI dataset, we did not consider the effects of other neurodegenerative pathologies, including cerebrovascular disease, α-synuclein, transactive response DNA-binding protein, argyrophilic grain pathology, and hippocampal sclerosis, on the progression model. Importantly, amyloid positivity might only be a contributing or incidental factor in some patients with dementia. This argument is mitigated to some degree by the fact that we included participants who progressed from MCI due to AD to AD dementia. Additionally, we found that the ADAS-cog 13 scores in some participants with CN and MCI improved over time. Although we controlled for LEs, we did not completely exclude the possibility that LEs might affect the disease progression to some degree.

Nevertheless, ADAS-cog 13 is the standard tool used in many clinical trials to assess AD, which makes our results more interpretable across studies than if we had used another instrument. Finally, our progression rate from NC to MCI (29.1%) was higher than has been observed in community-recruited older adults. For example, a greater risk of progression from NC to MCI was observed in clinically-recruited older adults (30% per year) than in community-recruited older adults (5% per year)^31^. The ADNI used identical recruitment mechanisms to those of typical trials, including advertising and recruitment from memory clinics. Although our data might not be representative of the general population, the recruitment and subject baseline characteristics were similar to those of a typical AD clinical trial.

In the current study, we found that our model of the progression to disease may help clinicians to predict where patients are in the disease course. In addition, it will help to predict how the disease course could vary by sex and APOE ε4 status when consulting with patients and predicting treatment effects. Understanding the natural history of AD and the rates of change of clinical phenotypes and biomarkers will facilitate specific appropriate interventions.

## Supplementary Information

Supplementary Information.

## Data Availability

All raw data are available on the ADNI website. Anonymized and statistical information of all the participants are available, upon reasonable request only among qualified investigators.
