# Supplementary material for: Disease progression modelling from preclinical Alzheimer’s disease (AD) to AD dementia
Source: Sci Rep. 2021 Feb 18;11:4168. doi: 10.1038/s41598-021-83585-3 (PMC7893024; doi:10.1038/s41598-021-83585-3)
Supplement: Supplementary file 1 — Supplementary Information. [file 41598_2021_83585_MOESM1_ESM.docx]

**Disease progression modelling from preclinical Alzheimer’s disease (AD) to AD dementia**

Soo Hyun Cho, MD, PhD^1,2*^, Sookyoung Woo, MS^3*^, Changsoo Kim, MD, PhD^4^, Hee Jin Kim, MD, PhD^1,5^, Hyemin Jang, MD, PhD ^1,5^, Byeong C. Kim, MD, PhD^2^, Si Eun Kim, MD ^6^, Seung Joo Kim, MD ^7^, Jun Pyo Kim,MD^1^, Young Hee Jung, MD, PhD ^8^, Samuel Lockhart, PhD^9^, Rik Ossenkoppele, PhD^10^, Susan Landau, PhD^11^, Duk L. Na, MD, PhD^1,5,12^, Michael Weiner, MD^13^, Seonwoo Kim, PhD^3†^, Sang Won Seo, MD, PhD^1,5,14,15,16†^, For the Alzheimer’s Disease Neuroimaging Initiative

**Authors’ affiliations:**

^1^Department of Neurology, Samsung Medical Center, Sungkyunkwan University School of Medicine, Seoul, Korea

^2^Department of Neurology, Chonnam National University Medical School, Chonnam National University Hospital, Gwangju, Korea

^3^Statistics and Data Center, Samsung Medical Center, Seoul, Korea

^4^Department of Preventive Medicine, Yonsei University College of Medicine, Seoul, Korea.

^5^Neuroscience Center, Samsung Medical Center, Seoul, Korea.

^6^Department of Neurology, Inje University College of Medicine, Haeundae Paik Hospital, Busan, Korea

^7^Department of Neurology, Gyeongsang National University School of Medicine and Gyeongsang National University Changwon Hospital, Changwon, Korea

^8^Department of Neurology, Myoungji hospital, Hanyang University, Goyangsi, Korea

^9^Internal Medicine - Gerontology and Geriatric Medicine, Wake Forest School of Medicine, Winston-Salem, NC, USA

^10^Department of Neurology and Alzheimer Center, VU University Medical Center, Neuroscience Campus Amsterdam, Amsterdam, the Netherland

^11^Helen Wills Neuroscience Institute, University of California, Berkeley, CA, USA

^12^Stem Cell & Regenerative Medicine Institute, Samsung Medical Center, Seoul, Korea

^13^Center for Imaging of Neurodegenerative Diseases, University of California, San Francisco, CA, USA

^14^Department of Health Sciences and Technology, SAIHST, Sungkyunkwan University, Seoul, Korea

^15^Samsung Alzheimer Research Center, Samsung Medical Center, Seoul, Korea

^16^ Department of Intelligent Precision Healthcare Convergence, Sungkyunkwan University School of Medicine, Suwon, Korea

*These individuals contributed equally to this article as co-first authors.

^†^These individuals contributed equally to this article as co-corresponding authors.

**Corresponding author 1**

**Sang Won Seo, MD, PhD**

Department of Neurology, Samsung Medical Centre, Sungkyunkwan University School of Medicine, 81 Irwon-ro, Gangnam-gu, Seoul, 06351, Korea.

Tel.: +82-2-3410-1233/-3599, Fax: +82-2-3410-0052, E-mail: sw72.seo@samsung.com

**Corresponding author 2**

**Seonwoo Kim, PhD**

Statistics and Data Centre, Samsung Medical Centre, 81 Irwon-ro, Gangnam-gu, Seoul, 06351, Korea.

Tel.: +82-2-3410-3661, Fax: +82-2-2148-7285, E-mail: [seonwoo.kim@samsung.com](mailto:seonwoo.kim@samsung.com)

**
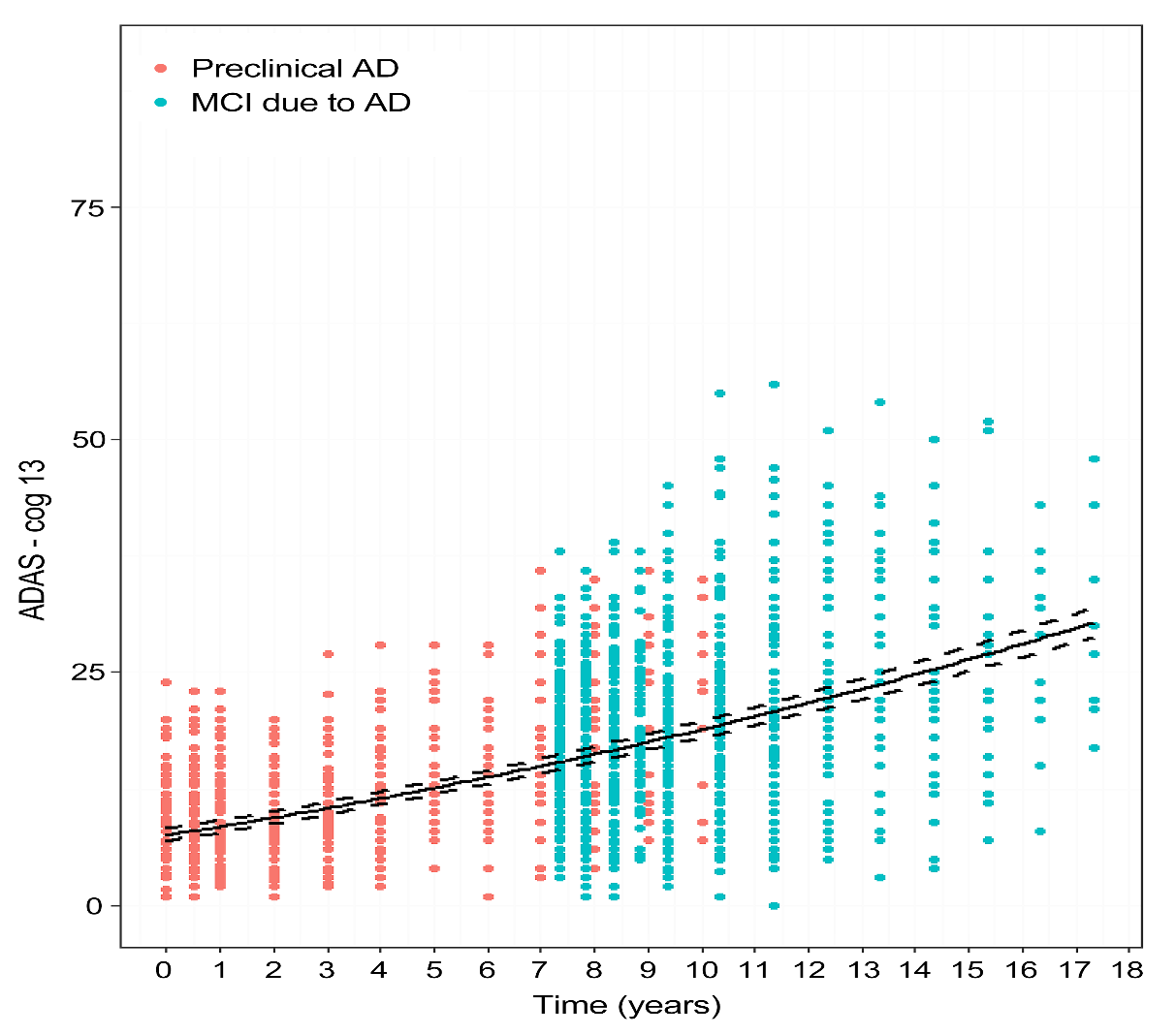
**

**Supplementary Fig. S1** Disease progression model from preclinical AD to AD dementia in frequency matched data with APOE ε4 allele carriage between preclinical AD and MCI due to AD cohorts. The curves present the estimated model (ADAS-cog 13 = (2.8836 + 0.0121× month)^2^ - 0.5). We calculated the time to convert from preclinical AD to MCI due to AD. From the matched data, ADAS-cog 13 score when the two cohorts to started to overlap was estimated to be 15.1 (95% CI=14.1–16.2) and the corresponding time was 7.4 years (88.2 months, 95% CI=77.0–99.4). Abbreviations: ADAS = Alzheimer's Disease Assessment Scale-cognitive subscale, MCI = mild cognitive impairment, AD **=** Alzheimer's disease, APOE **=** Apolipoprotein E**,** CI = Confidence interval

**
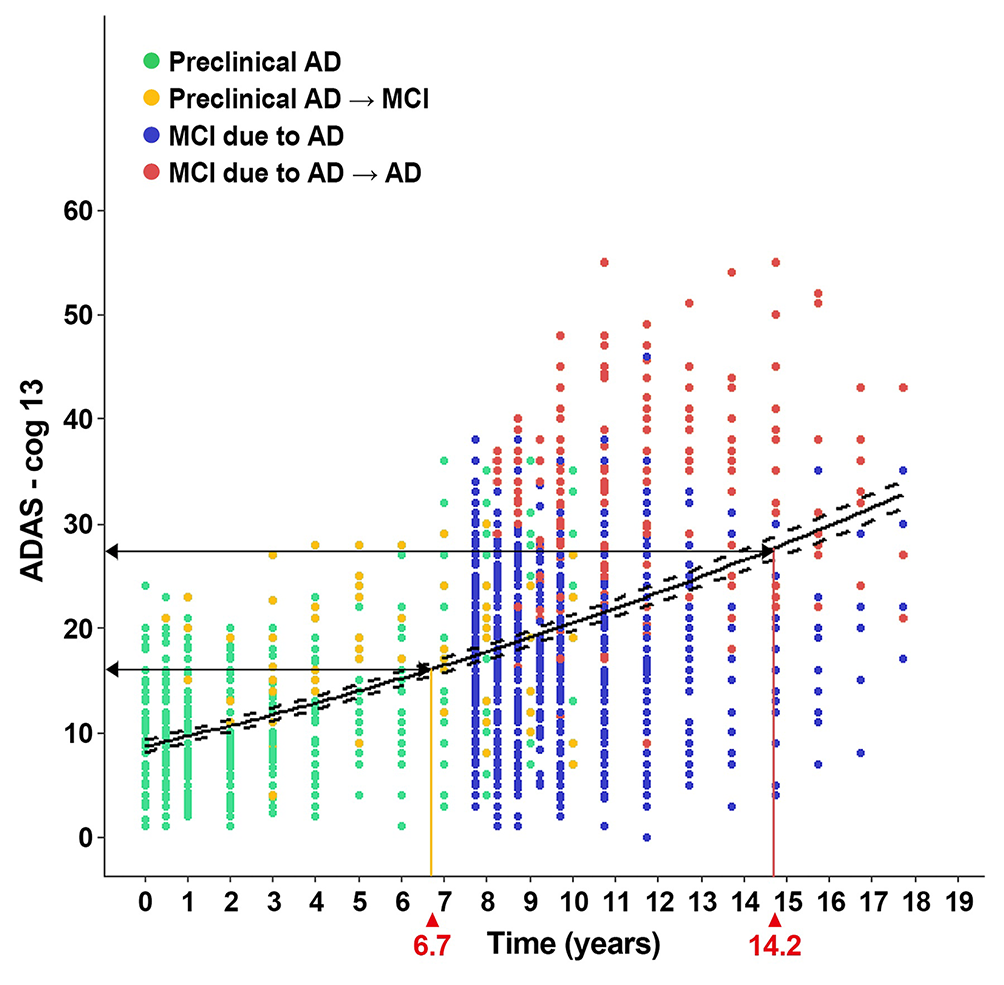
**

**Supplementary Fig. S2** Disease progression model from preclinical AD to AD dementia after correction for learning effects. The curves present the estimated model, ADAS-cog 13= (3.0312+0.0129×month)^2^ -0.5, and 95% CI corrected for learning effects from preclinical AD (green), converted MCI due to AD (yellow), MCI due to AD (blue) and converted AD dementia (red). Abbreviations: ADAS = Alzheimer's Disease Assessment Scale-cognitive subscale, AD = Alzheimer's Disease, MCI = mild cognitive impairment, CI = Confidence interval

**
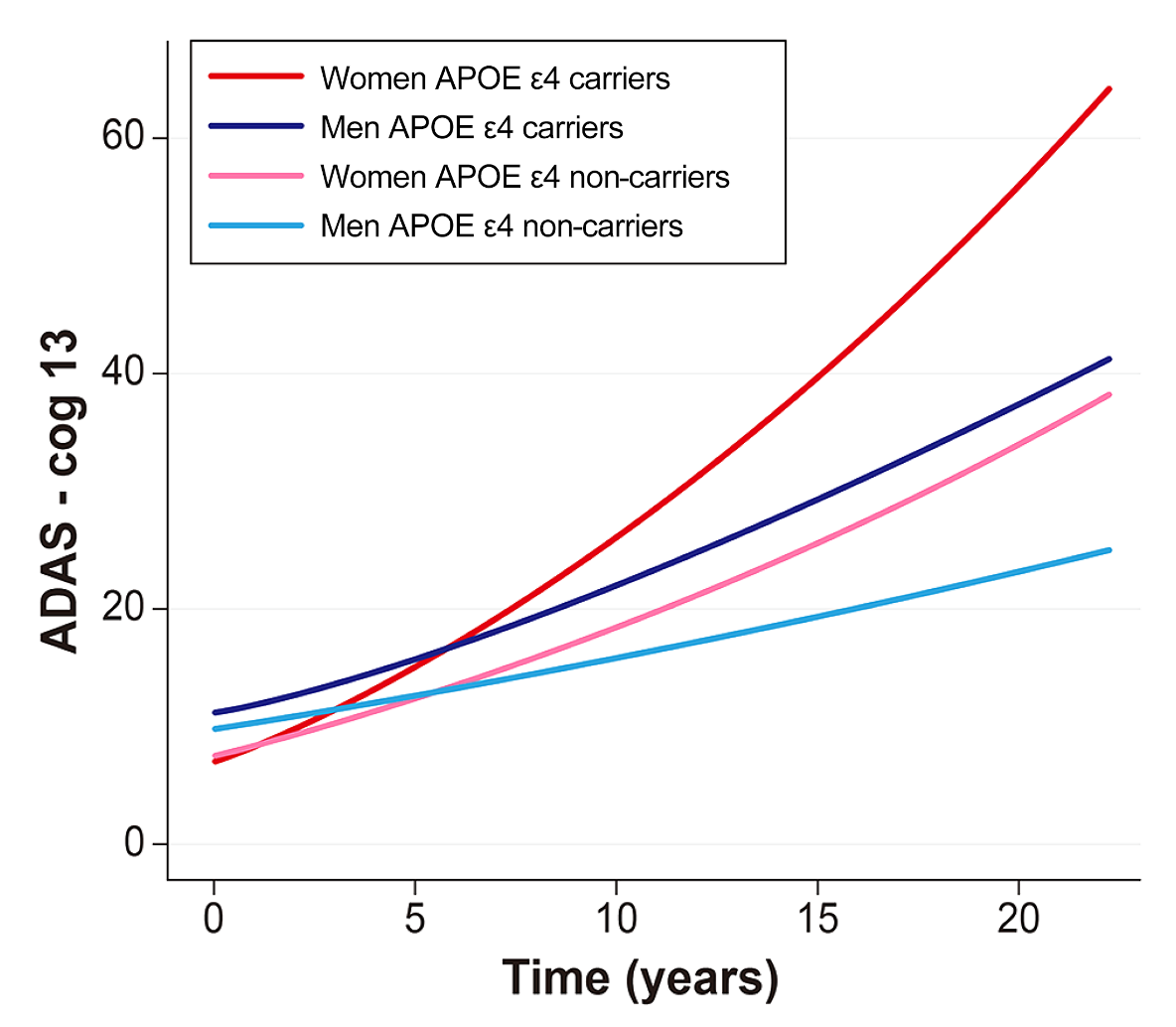
**

**Supplementary Fig. S3** Sex and APOE ε4 effects on disease progression course after correction for learning effects. We analysed the differences in cognitive decline with sex and APOE ε4 correcting for learning effect. Lines represent female APOE ε4 carriers (red), female APOE ε4 non-carriers (pink), male APOE ε4 carriers (dark blue) and male APOE ε4 non-carriers (light blue).

The estimated equation for each sex and APOE ε4 combination is as follows:

ADAS Cog-13 = (2.7748+0.0198×month)^2^-0.5 for female APOE ε4 carriers

= (2.8506+0.0126×month)^2^-0.5 for female APOE ε4 non-carriers

= (3.3618+0.0116×month)^2^-0.5 for male APOE ε4 carriers

= (3.2309+0.0068×month)^2^-0.5 for male APOE ε4 non-carriers

Abbreviations: ADAS = Alzheimer's Disease Assessment Scale-cognitive subscale, MCI = mild cognitive impairment, AD **=** Alzheimer's Disease, APOE = Apolipoprotein E

**Supplementary Table S1** Goodness of fit for the model without sex and APOE ε4, and with sex and APOE ε4

|  | Without sex and APOE ε4 | With sex and APOE ε4 |
| --- | --- | --- |
| -2 Res Log Likelihood | 4684.4 | 4641.3 |
| AIC | 4688.4 | 4645.3 |
| AICC | 4688.4 | 4645.3 |
| BIC | 4696.6 | 4653.5 |

Abbreviations: APOE = Apolipoprotein E, AIC = Akaike information criterion, BIC = Bayesian information criterion, AICC = AIC with correction for finite sample size.

**Supplementary Table S2 Learning effects estimated with six alternative models**

| Model | Preclinical AD | p value | MCI due to AD | p value |
| --- | --- | --- | --- | --- |
| 1) Intercept + Month + LE (Learning Effect) | -0.52 | <0.001 | -0.54 | <0.001 |
| 2) Model 1 adjusting for age, sex | -0.52 | <0.001 | -0.54 | <0.001 |
| 3) Intercept + Month + LE + Education level + Education level * Month | -0.52 | <0.001 | -0.54 | <0.001 |
| 4) Model 3 adjusting for age, sex | -0.52 | <0.001 | -0.54 | <0.001 |
| 5) Intercept + Month + LE + Education level + Education level * Month + Education level * LE | -0.48 | <0.001 | -0.44 | <0.001 |
|  | -0.50 | 0.85 | -0.57 | 0.07 |
|  | -0.55 | 0.61 | -0.56 | 0.12 |
| 6) Model 5 adjusting for age, sex | -0.48 | <0.001 | -0.44 | <0.001 |
|  | -0.51 | 0.81 | -0.57 | 0.07 |
|  | -0.55 | 0.61 | -0.56 | 0.12 |

We adjusted for learning effects (LE) because LE related to repeated measurements may obscure cognitive decline and delay detecting a conversion to MCI and AD. LE were estimated with 6 models according to covariates of age at baseline, sex, education level (12 years or less, 16 years or less, exceeded 16 years: 1^st^, 2^nd^ and 3^rd^ row of model 5 and 6) by using a linear mixed model considering subject effect as a random effect. ADAS-cog 13 score of -0.52 for preclinical AD and -0.54 for MCI due to AD were used because education level * LE (models 5 and 6) was not significant, so only models 1-4 were used. The model was created after the sqrt (ADAS13+0.5) transformation. Abbreviations: MCI = mild cognitive impairment, AD = Alzheimer's Disease, LE = learning effects
